# B7H3-targeting chimeric antigen receptor modification enhances antitumor effect of Vγ9Vδ2 T cells in glioblastoma

**DOI:** 10.1186/s12967-023-04514-8

**Published:** 2023-09-28

**Authors:** Yi Wang, Nan Ji, Yang Zhang, Junsheng Chu, Changcun Pan, Peng Zhang, Weiwei Ma, Xueguang Zhang, Jianzhong Jeff Xi, Mingze Chen, Yonghui Zhang, Liwei Zhang, Tao Sun

**Affiliations:** 1 Beijing Advanced Innovation Center for Biomedical Engineering, Beijing Advanced Innovation Center for Big Data-Based Precision Medicine, Beihang University, Beijing, 100191 China; 2 Department of Neurosurgery, Beijing Tiantan Hospital, Capital Medical University, Beijing, 100070 China; 3 China National Clinical Research Center for Neurological Diseases, Beijing, 100070 China; 4 Tsinghua-Peking Center for Life Sciences, State Key Laboratory of Membrane Biology, School of Pharmaceutical Sciences, Tsinghua University, Beijing, 100084 China; 5 Jiangsu Institute of Clinical Immunology, First Affiliated Hospital, Jiangsu Provincial Key Laboratory of Stem Cell and Biomedical Materials, Soochow University, Soochow University, Suzhou, 215000 China; 6 State Key Laboratory of Natural and Biomimetic Drugs, Institute of Molecular Medicine, Department of Biomedical Engineering, College of Engineering, Peking University, Beijing, 100871 China

**Keywords:** Vγ9Vδ2 T cells, Glioblastoma, B7-H3, BTN2A1, BTN3A1

## Abstract

**Background:**

Glioblastoma (GBM) is a highly aggressive primary brain tumor with a poor prognosis. This study investigates the therapeutic potential of human Vγ9Vδ2 T cells in GBM treatment. The sensitivity of different glioma specimens to Vγ9Vδ2 T cell-mediated cytotoxicity is assessed using a patient-derived tumor cell clusters (PTCs) model.

**Methods:**

The study evaluates the anti-tumor effect of Vγ9Vδ2 T cells in 26 glioma cases through the PTCs model. Protein expression of BTN2A1 and BTN3A1, along with gene expression related to lipid metabolism and glioma inflammatory response pathways, is analyzed in matched tumor tissue samples. Additionally, the study explores two strategies to re-sensitize tumors in the weak anti-tumor effect (WAT) group: utilizing a BTN3A1 agonistic antibody or employing bisphosphonates to inhibit farnesyl diphosphate synthase (FPPS). Furthermore, the study investigates the efficacy of genetically engineered Vγ9Vδ2 T cells expressing Car-B7H3 in targeting diverse GBM specimens.

**Results:**

The results demonstrate that Vγ9Vδ2 T cells display a stronger anti-tumor effect (SAT) in six glioma cases, while showing a weaker effect (WAT) in twenty cases. The SAT group exhibits elevated protein expression of BTN2A1 and BTN3A1, accompanied by differential gene expression related to lipid metabolism and glioma inflammatory response pathways. Importantly, the study reveals that the WAT group GBM can enhance Vγ9Vδ2 T cell-mediated killing sensitivity by incorporating either a BTN3A1 agonistic antibody or bisphosphonates. Both approaches support TCR-BTN mediated tumor recognition, which is distinct from the conventional MHC-peptide recognition by αβ T cells. Furthermore, the study explores an alternative strategy by genetically engineering Vγ9Vδ2 T cells with Car-B7H3, and both non-engineered and Car-B7H3 Vγ9Vδ2 T cells demonstrate promising efficacy in vivo, underscoring the versatile potential of Vγ9Vδ2 T cells for GBM treatment.

**Conclusions:**

Vγ9Vδ2 T cells demonstrate a robust anti-tumor effect in some glioma cases, while weaker in others. Elevated BTN2A1 and BTN3A1 expression correlates with improved response. WAT group tumors can be sensitized using a BTN3A1 agonistic antibody or bisphosphonates. Genetically engineered Vγ9Vδ2 T cells, i.e.,  Car-B7H3, show promising efficacy. These results together highlight the versatility of Vγ9Vδ2 T cells for GBM treatment.

**Supplementary Information:**

The online version contains supplementary material available at 10.1186/s12967-023-04514-8.

## Background

Glioblastoma (GBM) is the most common primary malignant brain tumor, accounting for 48.6% of all primary brain malignancies. It is classified as a grade IV glioma according to the World Health Organization (WHO) classification [1, 2]. Despite standard-of-care treatments such as surgical resection and radiotherapy, the median survival of GBM patients is only 15–20 months [3], and the prognosis remains poor with a 5 year survival rate of only 5.4% [4]. Thus, it is crucial to develop effective therapeutic strategies to enhance the prognosis of GBM patients.

Immunotherapy has shown promising results in the treatment of malignant tumors [5, 6]. However, the effectiveness of immunotherapy in treating GBM remains uncertain [7]. One of the most recent immunotherapy modalities for GBM is the adoptive transfer of γδ T lymphocytes expressing a T cell receptor (TCR) composed of γ and δ chains [8, 9]. In healthy peripheral blood, γδ T cells expressing the Vγ9Vδ2 chain account for 5–10% of CD3^+^ cells, representing the main subset of peripheral γδ T cells [10]. Zoledronic acid (ZOL) can inhibit the farnesyl diphosphate synthase in the mevalonate pathway, resulting in the accumulation of the upstream molecules isopentenyl pyrophosphate (IPP) and dimethylallyl pyrophosphate (DMAPP) [11]. Vγ9Vδ2 T cells detect changes in the IPP level of target cells through receptors such as TCR, thereby activating themselves and killing tumor cells. A breakthrough in understanding the possible mechanisms of IPP-mediated Vγ9Vδ2 T cells activation was the discovery that butyrophilin subfamily 2 member A1 (BTN2A1) and BTN3A1 are essential for Vγ9Vδ2 T cells activation [12, 13]. Activation of Vγ9Vδ2 T-cell receptor clonotypes can be induced by the BTN3A1-specific antibody 20.1 [14]. Like natural killer cells, Vγ9Vδ2 T cells express NKG2D receptors that can recognize MHC class I-related chain proteins A and B (MICA/B) on GBM cells [15–17]. Thus, Vγ9Vδ2 T cell immunotherapy is a promising therapeutic strategy for GBM treatment [18].

Despite significant progress in immunotherapy, chimeric antigen receptor (CAR)-T cell therapy based on αβ T cells has yet to achieve a breakthrough in treating solid tumors due to several limitations, such as T cell-associated toxicities, limited efficacy against solid tumors, antigen escape, poor trafficking and tumor infiltration, and the immunosuppressive microenvironment within the tumor [19, 20]. However, studies have shown that γδ T cells may serve as an alternative source of CAR-T cells [21, 22]. B7-H3 of the B7 family is highly expressed in over 70% of GBM specimens [23], and B7-H3-targeting immunotherapies are currently under clinical investigation in children and adults with refractory extracranial solid tumors and brain tumors (NCT02982941, NCT01391143, and NCT04185038) [24–26]. Therefore, we hypothesized that γδ T cells targeting B7-H3 could effectively suppress GBM development.

This study investigated the safety and antitumor effects of Vγ9Vδ2 T cells and Car-B7H3-γδT cells derived from healthy human peripheral blood mononuclear cells (PBMCs), and tested them against GBM cell lines, primary GBM cells, patient-derived tumor cell clusters (PTCs), and a mouse GBM model. We also conducted a preliminary investigation into the mechanism underlying the different responses of PTCs to Vγ9Vδ2 T cell therapy. Several methods were explored to enhance the anti-glioma ability of Vγ9Vδ2 T cells. The findings suggest that Car-B7H3-γδT cell immunotherapy has significant potential as a well-tolerated therapeutic approach for treating GBM.

## Methods

### Preparation of Vγ9Vδ2 T cells and car-B7H3-γδT cells

PBMCs were obtained from healthy subjects aged 18–50 years with a platelet count of ≥ 100 × 10^9^/L and activated partial thromboplastin time and prothrombin time within the normal range at Beijing Tiantan Hospital, China. Exclusion criteria: hematological diseases, steroid use within one month, and all malignancies. All participants provided signed informed consent. Vγ9Vδ2 T cells were expanded using a previously described method [27]. Briefly, the PBMCs was islated by Ficoll gradient (17544602, Cytiva, USA) from leukapheresis from healthy donors. GMP-compliant serum-free medium containing 200 U/mL IL2 and 2 mM L-glutamine (Gibco—Thermo Fisher Scientific, Waltham, MA, USA) was used to promote cell expansion. Zoledronic acid (5 μM, Sigma-Aldrich, St. Louise, MO, USA) was added to the medium on day 0 of cultivation and the cell concentration was 2 × 10^6^ cells/ml. The cells were incubated at 37 ℃, 5% CO_2_. The cell density was modified to 1 × 10^6^ cells/ml during the culture process every 2–3 days. On day 10, the number of Vγ9Vδ2 T cells was detected. The purity of Vγ9Vδ2 T cells was determined by flow cytometry. The cells were stained with anti-human TCR Vδ2-PerCP antibodies (BioLegend, clone: B6).

To generate Car-B7H3-γδT cells, we constructed a CAR from HIV-1-derived lentivirus vectors containing a mouse Ig Kappa leader sequence, CD8α hinge and transmembrane domains, and CD28, 4-1BB, and CD3ζ endo domains (FVPVFLPAKPTTTPAPRPPTPAPTIASQPLSLRPEACRPAAGGAVHTRGLDFACDIYIWAPLAGTCGVLLLSLVITLYCNHRNRSKRSRLLHSDYMNMTPRRPGPTRKHYQPYAPPRDFAAYRSRFSVVKRGRKKLLYIFKQPFMRPVQTTQEEDGCSCRFPEEEEGGCELRVKFSRSADAPAYQQGQNQLYNELNLGRREEYDVLDKRRGRDPEMGGKPRRKNPQEGLYNELQKDKMAEAYSEIGMKGERRRGKGHDGLYQGLSTATKDTYDALHMQALPPR). The configuration of the intracellular domain in the third generation of CAR CD28 can enhance the function of CAR-T cells. Additionally, the presence of the 4-1BB intracellular domain structure can contribute to the persistence of CAR-T cells [28, 29]. The scFv of the anti-B7-H3 antibody, consisting of heavy and light chains fused with a (GGGGS)_3_ linker, was inserted between the mouse Ig Kappa signal sequence and the CD8α hinge domain, resulting in the B7-H3-CD28/4-1BB/CD3z-CAR construct. A truncated version of the epidermal growth factor receptor (EGFRt) was co-expressed with a P2A cleavage peptide on the C terminal of the CAR construct. The CAR expression was monitored by measuring EGFRt expression. On day 10 after expansion, Car-B7H3-γδT cells were obtained by transfecting Vγ9Vδ2 T cells with B7-H3-CD28/4-1BB/CD3z-CAR, and the purity was characterized using flow cytometry.

### Human GBM tumor cell lines and cell culture

U-87MG, TJ905, and HTB15 cell lines (ATCC, Manassas, VA, USA) were maintained in DMEM (Gibco) supplemented with L-glutamine (Corning, Corning, NY, USA), 10% FBS, and 1% penicillin–streptomycin (Gibco) in a humidified atmosphere with 5% CO_2_ at 37 ºC. All cell lines were stably transfected with lentiviral luciferase vectors to generate U-87MG-Luc, TJ905-Luc, and HTB15-Luc cells.

### Human glioma tumor specimens, primary cells, and PTCs

This study was approved by the Ethics Committee of Beijing Tiantan Hospital (KY2014-021–02). Human glioma tumor specimens (n = 26) were obtained from patients enrolled in the Neurosurgery Clinical Information and Biobank Project of the Department of Brain Oncology, Beijing Tiantan Hospital, during tumor resection surgery. The patients were aged 18–72 years and were diagnosed with glioma based on histopathological examination. The tumor specimens were divided into three parts: one part was used to generate PTCs, an organoid tumor model for preclinical drug testing [30]. PTCs were cultured in RPMI medium (HyClone, Logan, UT, USA) and used for drug testing within 2 weeks after obtaining the tumor samples, as previously described [25, 30]. Another part was used to generate primary cells, which were cultured in 1% matrigel (356,243, BD, 4–12 h at 37 °C)-coated plates with serum-free DMEM (C11995500BT, Invitrogen), B27 (1:50), N2 (1:100), insulin (20 μg/mL), bFGF (20 ng/mL), EGF (20 ng/mL), PDGF-AB (20 ng/mL) (PeproTech). All primary cells were stably transfected with lentiviral luciferase vectors. The last part of the tumor specimens was used for RNA sequencing (RNA-Seq). The clinical information of the patients was summarized in Additional file 1: Table S1.

### Flow cytometry

The human GBM cell surfaces were stained with 10 µg/mL APC-labeled anti-human MICA/B mAb (#320908; Biolegend, San Diego, CA, USA), FITC-labeled anti-human BTN2A1 pAb (#orb499606; Biorbyt, UK), FITC-labeled anti-human BTN3A1 mAb (#14-2779-82; Invitrogen, Waltham, MA, USA), APC-labeled anti-human B7H3 (#351005; Biolegend), APC-labeled anti-human EGFR (#352906; Biolegend), or corresponding isotype control. The stained cells were analyzed using a FACS Calibur flow cytometer (Becton Dickinson, Franklin Lakes, NJ, USA).

### Coculture of T cells with GBM cells or PTCs

To assess the antitumor effects of Vγ9Vδ2 T cells or Car-B7H3-γδT cells, we incubated them with GBM cells or PTCs. Purified Vγ9Vδ2 T cells or Car-B7H3-γδT cells were centrifuged for 5 min at 300 *g* and resuspended in DMEM medium. T cells were then cocultured with target cells (U-87MG-Luc, TJ905-Luc, and HTB15-Luc cell lines) at different effective target (E:T) ratios of 0:1, 0.2:1, 0.5:1, 1:1, or 3:1 in the presence of interleukin 2 (IL-2) in a 96-well plate for 18–20 h at 37 °C. The luciferase activity was measured using a Bright-Lite luciferase detection system (#DD1204-01; Vazyme, Nanjing, China). The supernatant was collected to measure IFN-γ (#abs510007; Absin) and TNF-α (#abs510006; ABSIN) levels by ELISA.

To evaluate the antitumor effect of Vγ9Vδ2 T cells on PTCs (n = 26), Vγ9Vδ2 T cells were cocultured with each sample at a ratio of 3:1. The antitumor effect was assessed by measuring the diameter changes of PTCs at 8 h after incubation using a confocal high-content imaging microscope (Opera Phenix). The antitumor effect was calculated as (the diameter of PTCs after coculture/the diameter of PTCs before coculture) × 100%. Patients were categorized into two types according to the antitumor effect: those with an antitumor effect ≥ 50% were defined as having a stronger antitumor effect (SAT), while those with an antitumor effect < 50% were defined as having a weaker antitumor effect (WAT).

### ZOL or BTN3A1 agonistic monoclonal antibody stimulation

To investigate whether the addition of ZOL or BTN3A1 agonistic monoclonal antibody could enhance the killing effect of Vγ9Vδ2 T cells against WAT primary glioma cells (TT-LS, TT-YZJ, TT-ZLH, TT-XJ, TT-SX, and TT-HCL), Vγ9Vδ2 T cells were co-cultured with insensitive primary glioma cells at a 1:1 ratio for 18 to 20 h. The co-cultures were supplemented with ZOL (at a working concentration of 5 μM, Sigma), activating BTN3A1 agonistic monoclonal antibody (eBioBT3.1 (20.1, BT3.1), eBioscience™), or a control. The luciferase activity was then measured.

U87-B7H3-KO (B7H3 knocked out) cells stably transfected with lentiviral luciferase vectors were kindly provided by Dr. Xueguang Zhang from Soochow University. To assess whether Car-B7H3-γδT cells retained their function as Vγ9Vδ2 T cells, Car-B7H3-γδT and Vγ9Vδ2 T cells were co-cultured with U87-B7H3-KO cells in 1:1 ratio for 18 to 20 h in the presence or absence of ZOL (5 μM working concentration, Sigma). The luciferase activity was measured.

### Orthotopic xenograft glioma model

Immunodeficient NSG mice (NOD.Cg-Prkdcscid Il2rgtm1Wjl/SzJ, 6–8 weeks old, weighing 20–25 g) were obtained from Sperf Biotech, Beijing, China, and housed under specific-pathogen-free conditions at 22 ± 1 ºC, 50 ± 1% humidity, with a 12/12 h light/dark cycle and free access to water and a Chow diet. All animal procedures were conducted following the guidelines of the Beijing Neurosugical Institute Laboratory Animal Welfare and Ethics Committee (BNI Approval Number: 202202002) and in accordance with the AAALAC and IACUC Guidelines. The study was approved by the Beijing Regional Ethics Committee.

Each mouse received an intracranial injection of 5 μL phosphate-buffered saline (PBS) containing 10^5^ U-87MG-Luc cells at a rate of 1 μL/min using a stereotactic tumor establishment device. The coordinates for the injection were 2 mm to the right from the bregma and 3.5 mm depth. Six to nine days after injection, mice were intraperitoneally injected with 150 ng/g D-luciferin (#122,796; PerkinElmer, Waltham, MA, USA). Tumor formation was observed and imaged using an IVIS system (Caliper Life Sciences, Mountain View, CA, USA) after 10 min of injection. The IVIS data was analyzed using the Living Image software (Caliper Life Sciences). Tumor formation was also confirmed by MRI examination.

The tumor-bearing mice were randomly divided into three groups: Vγ9Vδ2 T cell group, Car-B7H3-γδT cell group, and control group (n = 5/group). Each mouse received an intracranial injection of 5 × 10^6^ Vγ9Vδ2 T cells or Car-B7H3-γδT cells or PBS into the lateral ventricle (0.5 mm behind bregma, 1 mm left, 3 mm depth). The mice were then intraperitoneally injected with 100 μL of IL-2 (5000 IU) twice, at 3 day intervals. The control group was injected with PBS. The mice were weighted twice a week to monitor body weight changes. Bioluminescence intensities of the tumors were measured once a week.

### Cytokine multiplex assay

Cytokine multiplex assay was conducted to evaluate the safety of T cell therapy in mice. Blood samples were collected from the retrobulbar sinus, and serum was obtained by centrifuging the blood samples at 5000 *g* for 5 min and stored at − 80 °C until use. Serum cytokine levels were measured using a cytokine array kit (#QAH-INF-3–2, QAH-GF-1–2; RayBiotech, Peachtree Corners, GA, USA) following the manufacturer’s instructions. The fluorescence intensities were measured using an Axon scanner 4000B and GenePix software. The results were analyzed using a RayBio analysis tool. For each cytokine, the mean fluorescence intensity was calculated, and the z-score was generated. A heatmap of the z-scores ranging from − 3 to 3 was generated using ggplot2 v3.2.1.

### Immunohistochemistry (IHC) and multiplex immunofluorescence staining (MIS)

Human glioma tissue samples were fixed with 4% paraformaldehyde, embedded in paraffin, and cut into 5 µm-thick sections. The tissue sections were deparaffinized, rehydrated, and underwent heat-induced epitope repair retrieval. Then, the sections were incubated with anti-BTN2A1 (1:200; #orb499606; Biorbyt) or anti-BTN3A1 (1:400; #CAB10288; Genie) antibodies at 4 °C, followed by incubation with a secondary antibody (1:1000; #ZB-2301; ZSGB-BIO, Beijing, China) for 1 h. DAB peroxidase substrate solution staining, hematoxylin blue staining, and differentiation reverse blue staining were performed. The slides were scanned using NanoZoomer 2.0 HT (Hamamatsu Photonics KK, Hamamatsu, Japan). The positive rate was calculated as (the number of positive cells/the number of total cells) × 100%. An IHC score was calculated as the positive rate × staining intensity. An IHC score > 10 was considered positive.

MIS was conducted in glioma tissue samples from mice to evaluate the infiltration of Vγ9Vδ2 T cells into tumors using an OPAL^™^ 7-color Manual IHC kit (#NEL811001 KT; Akoya Bioscience, Marlborough, MA, USA) following the manufacturer’s protocol. The detailed experimental procedures and conditions are summarized in Additional file 1: Table S2.

### RNA-Seq

RNA-Seq was performed using glioma tissue samples from patients. DESeq2 (version 1.32.0) was used to identify differentially expressed genes (DEGs) with |fold change|> 2 and adjusted *P*-value < 0.05. Gene set enrichment analysis (GSEA) was performed on hallmark gene sets from the Molecular Signatures Database (MSigDB, v7.2) to analyze the enrichment of biological pathways.

### Statistical analysis

Statistical analysis was performed using the SPSS software (version 24.0; SPSS, Chicago, IL, USA). Categorical data were compared using chi-squared test or Fisher’s exact test. Continuous data were compared using Student’s t-test or non-parametric test. Kaplan–Meier analysis was used to estimate overall survival. A comparison between two groups was conducted using the Mann–Whitney U-test. A two-tailed *P*-value < 0.05 was considered significant. The plots were generated using Prism 6 (GraphPad Software, San Diego, CA, USA) and the R2 package.

## Results

### Vγ9Vδ2 T cells inhibit GBM cell proliferation in vitro

**Fig. 1:**
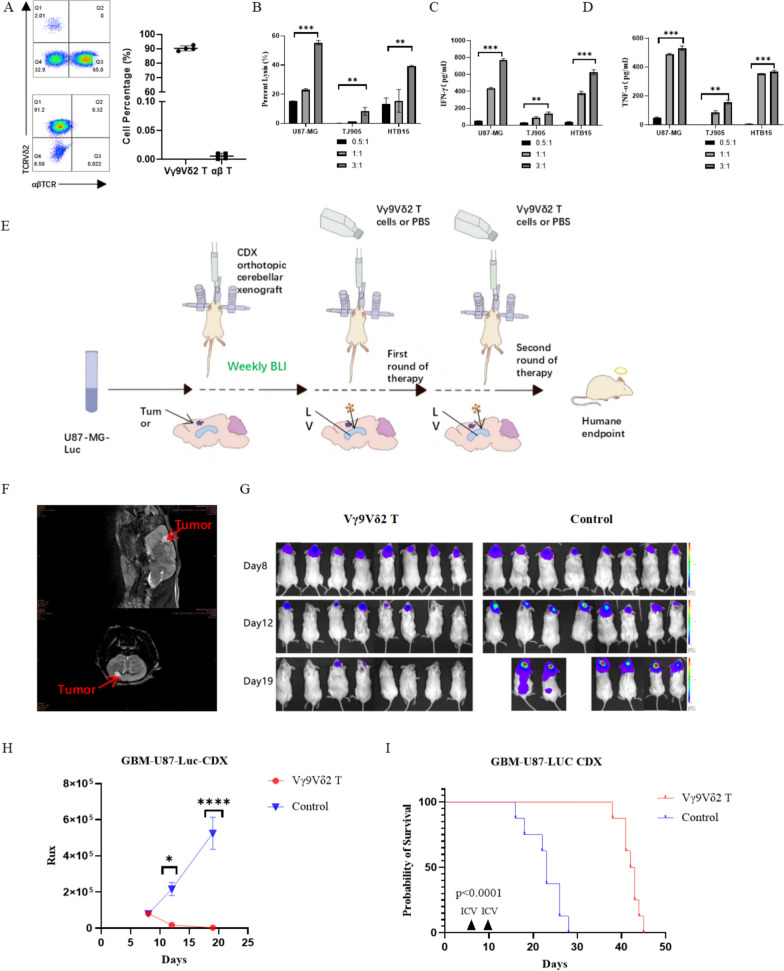
Preparation of Vγ9Vδ2 T cells and their inhibition of glioblastoma in vitro and in vivo. **A** A total of 2 × 10^6^ peripheral blood mononuclear cells (PBMCs) were obtained from healthy human donors and expanded using GMP-compliant serum-free medium containing bisphosphonate compounds and factors. Flow cytometry was performed to determine the purity of Vγ9Vδ2 T cells on day 10 after cultivation (n = 4). **B** Vγ9Vδ2 T cells were incubated with U-87MG-Luc, TJ905-Luc, or HTB15-Luc cells at different effective target (E:T) ratios of 0:1, 0.5:1, 1:1, or 3:1 in the presence of IL-2. The cell viability of GBM cells was determined by measuring luciferase activity after 18–20 h. Data are expressed as the mean ± standard deviation (SD). *P < 0.05, **P < 0.01, ***P < 0.001. **C, D** ELISA was performed to measure IFN-γ and TNF-α levels in the culture medium. Data are expressed as the mean ± SD. *P < 0.05, ***P < 0.001, ****P < 0.0001; n = 3. **E** An NSG mouse GBM model was established by intracerebroventricular injection of U87-MG-Luc cells. Mice were treated with Vγ9Vδ2 T cells or PBS (n = 8). A workflow diagram is shown. **F** MRI imaging was performed to visualize the tumor (red arrow). **G** Bioluminescence images of the mouse brain were taken on day 8, 12, and 19 after injection. **H** Bioluminescence intensity was measured to examine tumor growth. Data are expressed as the mean ± SD. ****P < 0.0001. **I** Kaplan-Meier survival analysis was performed

To expand Vγ9Vδ2 T cells from healthy human PBMCs more rapidly and efficiently, we applied serum-free media containing amino bisphosphonates as previously described [27]. We obtained Vγ9Vδ2 T cells with a purity of around 90% within 10 days (Fig. 1A**)**. To evaluate the cytotoxicity of Vγ9Vδ2 T cells, we co-cultured Vγ9Vδ2 T cells with GBM-Luc cells at different E:T ratios in the presence of IL-2. As shown in Fig. 1B, Vγ9Vδ2 T cells inhibited GBM cell proliferation in a dose-dependent manner, as evidenced by decreased luciferase activity of the cocultures (*P* < 0.05). The most prominent antitumoral activity of Vγ9Vδ2 T cells was observed in U87-MG-Luc cells. Additionally, Vγ9Vδ2 T cells produced IFN-γ and TNF-α when cocultured with GBM cell lines (all *P* < 0.0001) (Fig. 1C, D). These results suggest that Vγ9Vδ2 T cells inhibit GBM cell proliferation possibly by producing IFN-γ and TNF-α.

### Vγ9Vδ2 T cell therapy suppresses GBM growth and improves prognosis in mice

We established a GBM mouse model by injecting U87-MG-Luc cells into the frontal cortex of mice, followed by Vγ9Vδ2 T cell therapy (Fig. 1E). The cell line-derived xenograft model was confirmed by MRI imaging **(**Fig. 1F**)**, bioluminescence analysis **(**Fig. 1G**),** and hematoxylin and eosin (H and E) staining (Additional file 1: Figure S1). The tumor-bearing mice were randomly treated with Vγ9Vδ2 T cells or PBS, infused back into the lateral cerebral ventricle. We found that compared with PBS vehicle, Vγ9Vδ2 T cell therapy dramatically suppressed GBM tumor growth in mice (Fig. 1G, H**)** and improves survival (42 days vs. 23 days, *P* < 0.0001) (Fig. 1I) without changing the body weights of mice. These data suggest that Vγ9Vδ2 T cells can effectively suppress GBM growth and improve prognosis in vivo.

### The anti-tumor activities of Vγ9Vδ2 T cell therapy vary depending on the PTC being treated

**Fig. 2:**
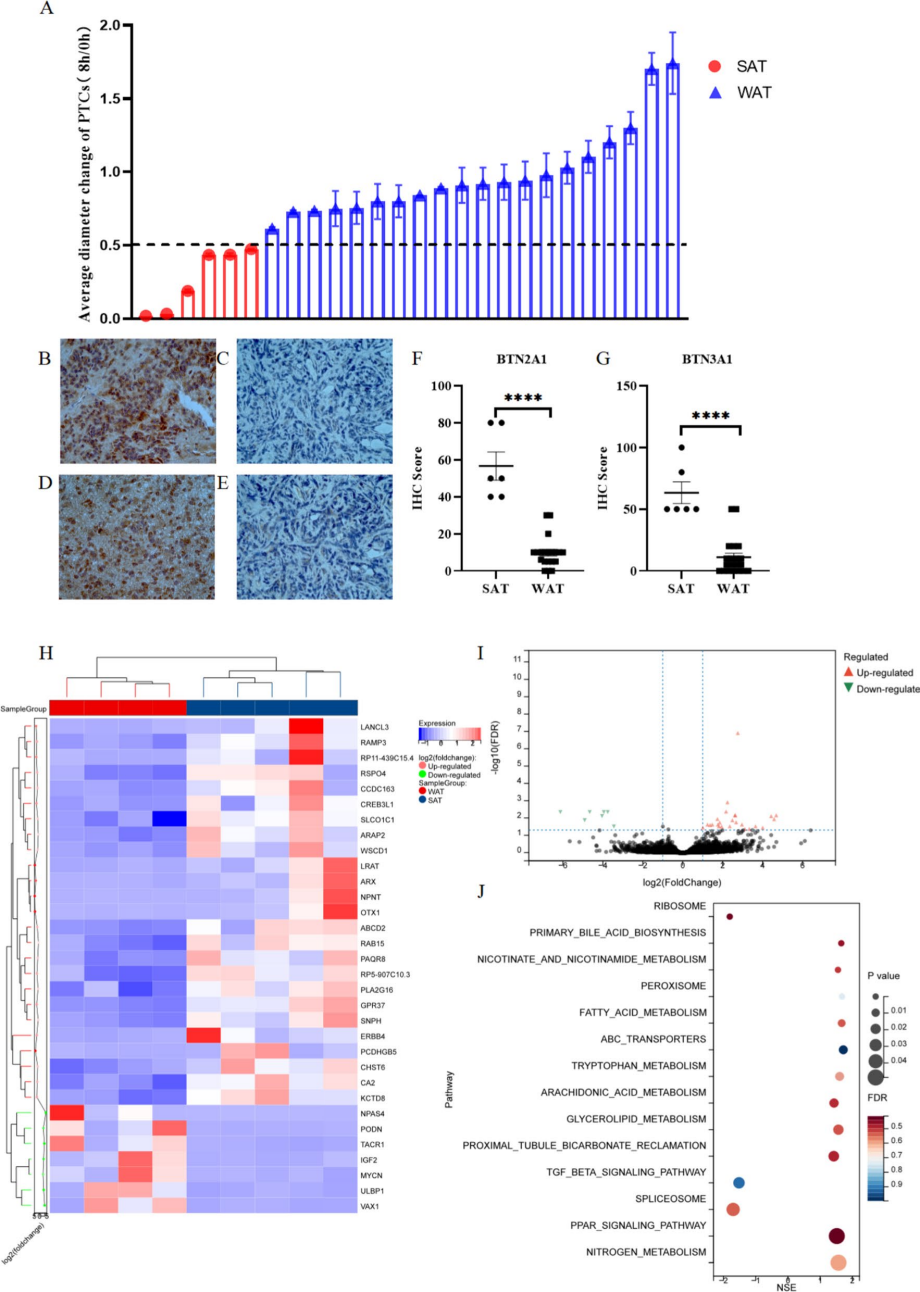
Vγ9Vδ2 T cell therapy exhibited different anti-tumor activities on patient-derived tumor cell clusters (PTCs) **A** GBM patients (n = 26) were categorized into two groups according to the antitumor effects of Vγ9Vδ2 T cells on matched PTCs by co-culture killing assay: stronger anti-tumor effect (SAT; n = 6) and weaker anti-tumor effect (WAT; n = 20) groups **B–E** Immunohistochemical (IHC) staining was performed to detect BTN2A1 **B**, **C** and BTN3A1 **D**, **E** expression in tumor tissue samples from patients in the SAT **B**, **D** and WAT **C**, **E** groups. **F, G** IHC scores were calculated. **H** RNA sequencing was performed on glioma tissue samples from patients. A heatmap of differentially expressed genes (DEGs; |fold change|> 2 and adjusted *P*-value < 0.05) in patients with SAT versus those with WAT is shown. **I** A volcano plot of the DEGs is displayed. **J** KEGG analysis of the DEGs was conducted. The top 14 enriched pathways are shown

PTC is an emerging preclinical drug-testing model [30]. To evaluate the clinical significance of Vγ9Vδ2 T cell therapy, we co-cultured Vγ9Vδ2 T cells with different PTCs (Additional file 1: Table S1**)** at a 3:1 ratio in the presence of IL-2 for 8 h. We observed significant differences in the responses to Vγ9Vδ2 T cells among the PTCs, with SAT responses in six PTCs (1 grade II glioma, 1 grade III glioma, and 4 GBM) and WAT responses in twenty PTCs (6 grade II glioma, 4 grade III glioma, and 10 GBM) (Fig. 2A). No significant correlation was observed between the antitumor effect of Vγ9Vδ2 T cells and tumor grade (*P* > 0.05) (Additional file 1: Table S3). This finding suggests that Vγ9Vδ2 T cell therapy might be effective for some GBM patients, but additional factors beyond tumor grade may affect the therapy's effectiveness.

### BTN2A1 and BTN3A1 expression correlates with the antitumor activity of Vγ9Vδ2 T cells

Since Vγ9Vδ2 T cells can recognize tumor cells expressing BTN2A1 and BTN3A1 [12, 31, 32], we compared the expression of these two molecules in different GBM cell lines by flow cytometry and matched tumor samples of PTCs using IHC staining. Our results showed that BTN2A1 and BTN3A1 were highly expressed in U87-MG cell line (Additional file 1: Figure S2A) and the SAT group **(**Fig. 2B, D**),** compared to the WAT group (Fig. 2C, E**)**. Furthermore, the BTN2A1 and BTN3A1 IHC scores were significantly higher in the SAT group than in the WAT group **(**Fig. 2F, G). Specifically, all samples in the SAT group were double-positive for BTN2A1 and BTN3A1, while only a small fraction of the WAT group samples were BTN2A1-positive (15%), BTN3A1-positive (25%), or double-positive (15%) (Table 1). No BTN2A1 or BTN3A1 expression was detected in tumor-adjacent tissue or normal brain tissue. In addition, no significant correlation was observed between BTN2A1/BTN3A1 protein expression and WHO grade (*P* > 0.05) (Table 2). These results suggest that Vγ9Vδ2 T cells may inhibit GBM growth by targeting BTN2A1 and BTN3A1 on GBM tumor cells.

**Table 1:** Immunohistochemistry analysis of BTN2A1 and BTN3A1 expression in tumor tissue samples of glioma patients with different responses to Vγ9Vδ2 T therapy

Antitumor effect on PTCs	Total patients (n)	BTN2A1 Expression			BTN3A1 Expression			BTN2A1 + BTN3A1 expression			
		Positive (n)	Negative (n)	Positive Rate (%)	Positive (n)	Negative (n)	Positive Rate (%)	Positive (n)	Negative (n)	Positive Rate (%)	P value*
SAT	6	6	0	100	6	0	100	6	0	100	0.00012
WAT	20	3	17	15	5	15	25	3	17	15	

*SAT* stronger anti-tumor effect, *WAT* weaker anti-tumor effect

^a^Chi-squared tests

**Table 2:** Immunohistochemisty analysis of BTN2A1 and BTN3A1 expression in tumor tissue samples from patients with different grades of glioma

WHO grade	Total Patients (n)	BTN2A1 Expression		Positive Rate (%)	BTN3A1 Expression		Positive Rate (%)	*P* Value
		Positive (n)	Negative (n)		Positive (n)	Negative (n)		
II	7	2	5	29	2	5	29	
III	5	1	4	20	2	3	40	> 0.05
IV	14	6	8	43	7	7	50	

To explore the molecular mechanisms involved in the antitumor activity of Vγ9Vδ2 T cells in GBM, we performed RNA-seq to identify DEGs between SAT and WAT tissue samples and used GSEA to analyze the enriched pathways. Our results revealed that Vγ9Vδ2 T cell response was mainly related to fatty acid metabolism, primary bile acid biosynthesis, nicotinate and nicotinamide metabolism, peroxisome, ABC transporters, tryptophan metabolism, arachidonic acid metabolism, glycerolipid metabolism, proximal tubule bicarbonate reclamation, TGF beta signaling pathway, and PPAR signaling pathway **(**Fig. 2H–J**)**. These data suggest that lipid metabolism and glioma inflammatory response may play important roles in the antitumor effect of Vγ9Vδ2 T cells on GBM.

### ZOL or BTN3A1 agonistic antibody enhances the antitumor activity of Vγ9Vδ2 T cells in glioma

**Fig. 3:**
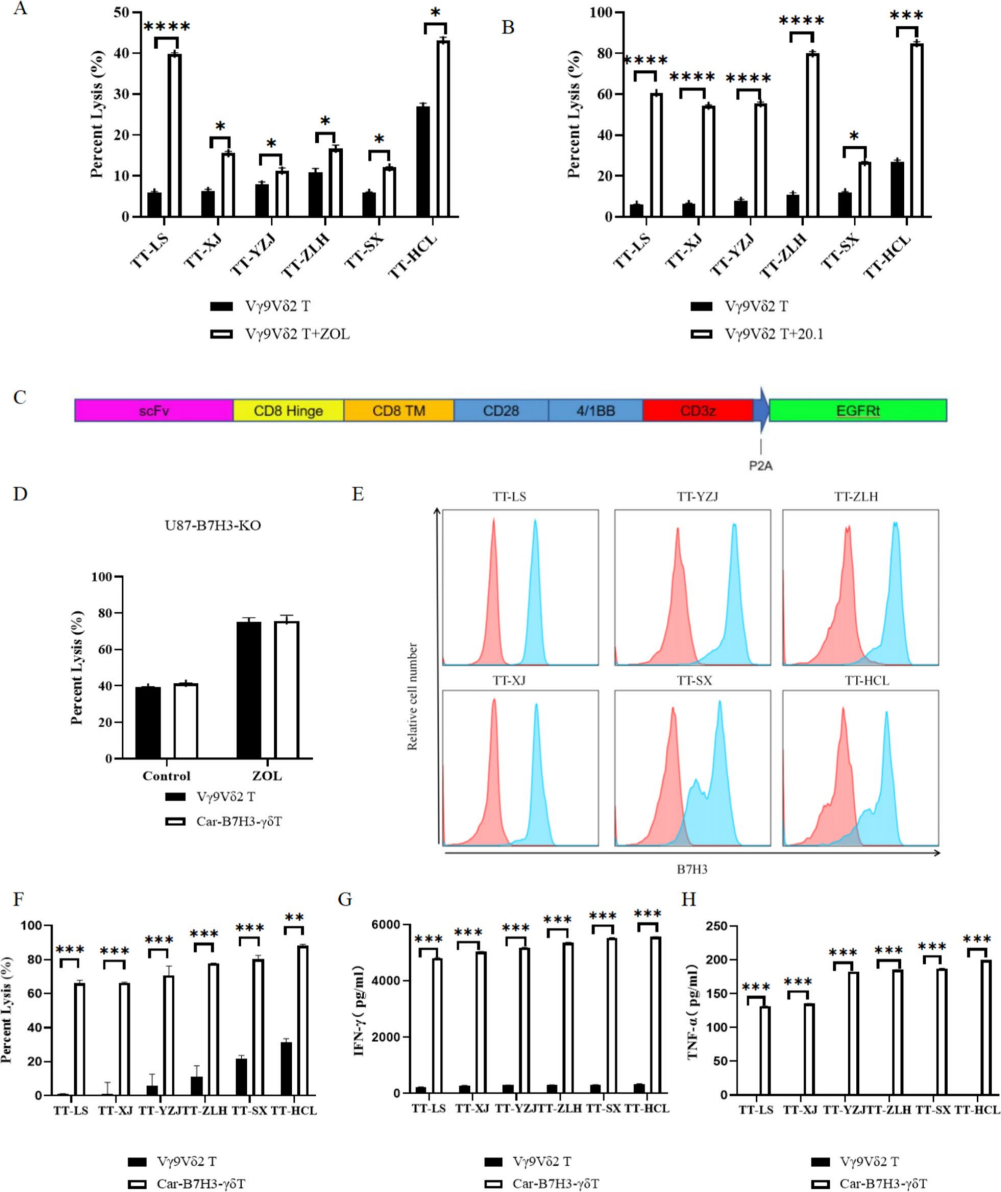
The strategy for enhancing Vγ9Vδ2 T cell therapy in the treatment of glioma in the WAT group. Vγ9Vδ2 T cells were incubated with WAT primary glioma cells (TT-LS-luc, TT-YZJ-luc, TT-ZLH-luc, TT-XJ-luc, TT-SX-luc, and TT-HCL-luc) at E:T ratios of 1:1 in the presence or absence of ZOL (5 μM) or BTN3A1 agonistic antibody (BTN3A1 20.1, 1 µg/mL) for 18–20 h. The killing of Vγ9Vδ2 T cells against WAT primary glioma cells was significantly enhanced after ZOL stimulation **A** or BTN3A1 agonistic antibody stimulation **B**. Data are expressed as mean ± SD. *P < 0.05, **P < 0.01, ***P < 0.001, ****P < 0.0001. Car-B7H3-γδT cells had stronger and wider anti-glioma ability than parental Vγ9Vδ2 T cells in vitro. **(C)** CAR construct is shown. (**D**)Vγ9Vδ2 T cells and Car-B7H3-γδT cells were co-cultured with U87-B7H3-KO cells in a 1:1 ratio for 18–20 h in the presence or absence of ZOL (5 μM). Luciferase activity was measured. Data are expressed as the mean ± SD. **E** Flow cytometry analysis was performed to determine B7-H3 expression in WAT primary glioma cells. The expression of B7-H3 protein was higher than 90%. **F** Vγ9Vδ2 T cells and Car-B7H3-γδT cells were incubated with WAT primary glioma cells at E:T ratios of 1:1 for 18–20 h. Luciferase activity was measured. Data are expressed as the mean ± SD. *P < 0.05, **P < 0.01, ***P < 0.001. **G, H** The culture medium was collected, and ELISA was performed to measure IFN-γ and TNF-α levels. Data are expressed as the mean ± SD. *P < 0.05, ***P < 0.001, ****P < 0.0001; n = 3

To investigate the potential of enhancing the antitumor activity of Vγ9Vδ2 T cells in unsensitive glioma, we co-cultured Vγ9Vδ2 T cells with WAT primary glioma cells (TT-LS-luc, TT-YZJ-luc, TT-ZLH-luc, TT-XJ-luc, TT-SX-luc, and TT-HCL-luc) at E:T ratios of 1:1 in the presence or absence of ZOL or BTN3A1 agonistic antibody. Our results showed that the killing of Vγ9Vδ2 T cells against primary glioma cells was significantly enhanced after ZOL (Fig. 3A) or BTN3A1 agonistic antibody stimulation (Fig. 3B). These findings suggest that ZOL and BTN3A1 agonistic antibody can stimulate glioma cells to enhance their sensitivity to Vγ9Vδ2 T cells. However, since the safety of these two drugs has not been confirmed for intracerebroventricular injection, they cannot be used in clinical treatment of glioma at present.

### Car-B7H3-γδT cells maintain a functional endogenous TCR

To address this issue, we transfected Vγ9Vδ2 T cells with plasmids (Fig. 3C**)** containing the scFv of the anti-B7-H3 antibody and obtained Car-B7H3-γδT cells with a purity of around 69.5% (Additional file 1: Figure S2B). Because the Vγ9Vδ2 TCR is activated by accumulation of phosphoantigens induced by bisphosphonates such as ZOL, we tested whether Car-B7H3-γδT cells maintained the functionality of their endogenous TCR [33]. When Vγ9Vδ2 T cells or Car-B7H3-γδT cells were co-cultured with U87-B7H3-KO at an E:T ratio of 1:1 and stimulated with ZOL, both types of T cells showed enhanced cytotoxic effects (Fig. 3D). These results suggest that Car-B7H3-γδT cells can still be stimulated by ZOL to enhance their antitumor effect, similar to Vγ9Vδ2 T cells.

### Car-B7H3-γδT cells exhibit stronger anti-glioma activity than parental Vγ9Vδ2 T cells in vitro

To evaluate the potential of Car-B7H3-γδT lymphocytes in clinical settings, we examined the expression of B7-H3 in six primary cell lines generated from the WAT group. All six cell lines were IDH wild type, among which three had unmethylated MGMT promoters (TT-LS, TT-YZJ, and TT-ZLH) and three had methylated MGMT promoters (TT-XJ, TT-SX, and TT-HCL). Flow cytometry results showed that B7-H3 was highly expressed in all six primary cell lines (positive rate > 90%) (Fig. 3E). Similarly, flow analysis revealed that B7-H3 was highly expressed on the surface of U-87MG, TJ905, and HTB15 cells (Additional file 1: Figure S2C).

We co-cultured Vγ9Vδ2 T and Car-B7H3-γδT cells with the six primary cell lines at different E:T ratios in the presence of IL-2. As shown in Fig. 3F, at the same E:T ratio (1:1), Car-B7H3-γδT cells significantly inhibited the proliferation of all six primary cell lines, while Vγ9Vδ2 T cells were insensitive (all* P* < 0.05). Additionally, Car-B7H3-γδT cells produced more IFN-γ and TNF-α when cocultured with primary WAT cells (all *P* < 0.05) (Fig. 3G, H). Similarly, at the same E:T ratio, Car-B7H3-γδT cells exhibited a more potent inhibitory effect on GBM cell line proliferation than Vγ9Vδ2 T cells (*P* < 0.05) (Additional file 1: Figure S2D). Moreover, Car-B7H3-γδT cells secreted more cytokines than Vγ9Vδ2 T cells (*P* < 0.05) **(**Additional file 1: Figure S2E). These data suggest that Car-B7H3-γδT cells exhibit stronger and wider anti-glioma activity than parental Vγ9Vδ2 T cells in vitro.

### Car-B7H3-γδT cells demonstrate stronger inhibition of GBM growth in mice compared to Vγ9Vδ2 T Cells

**Fig. 4:**
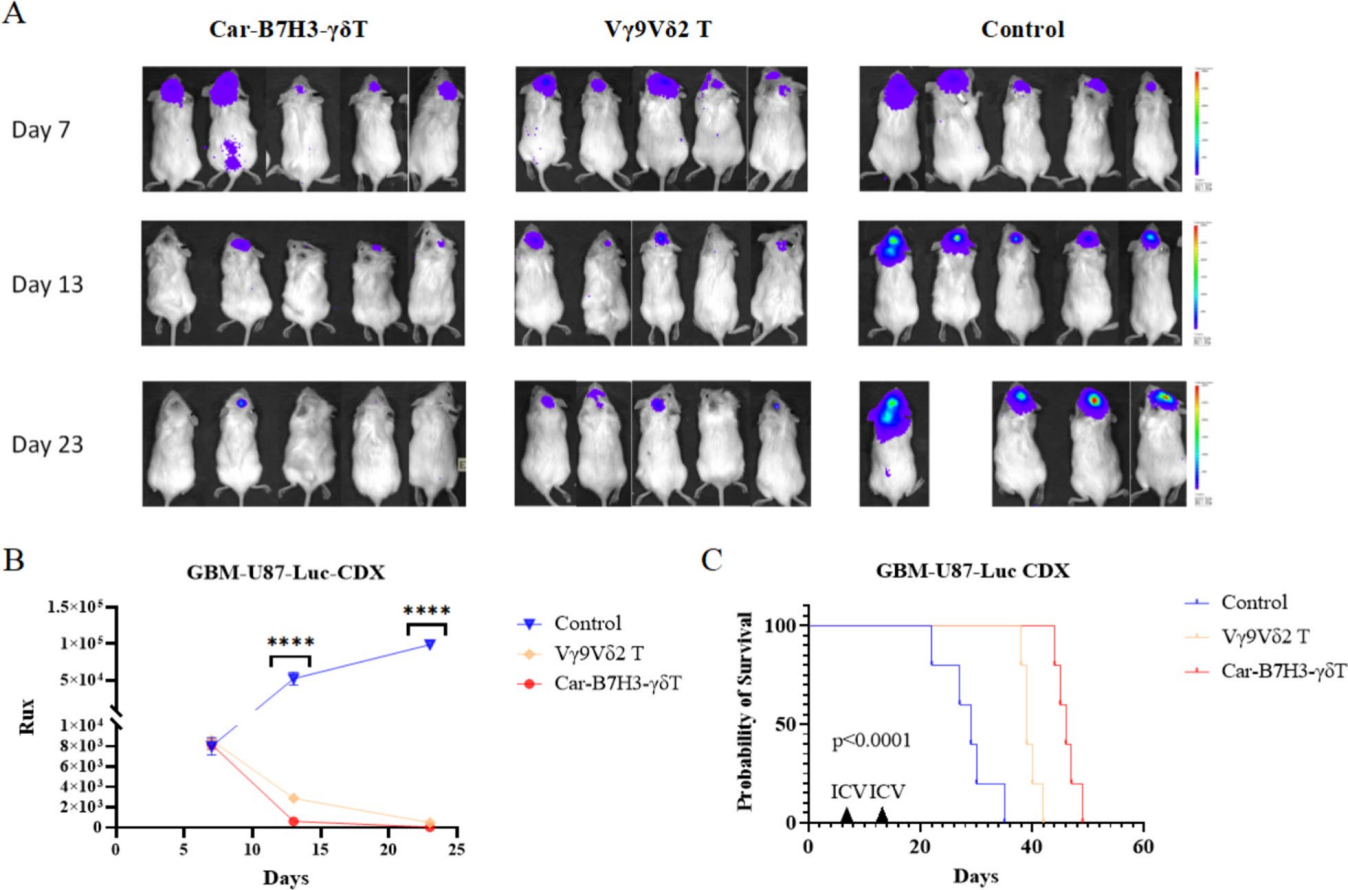
The anti-glioma ability of Car-B7H3-γδT cells was stronger than that of parental Vγ9Vδ2 T cells in vivo **A** NSG mouse GBM models were treated with Vγ9Vδ2 T cells, Car-B7H3-γδT cells, or PBS (n = 5). Bioluminescence images of the mouse brain were taken on day 7, 13, and 23 after injection. **B** Bioluminescence intensity was measured to examine tumor growth. Data are expressed as mean ± SD. ****P < 0.0001. **C** Kaplan-Meier survival analysis was performed

To evaluate the antitumor effects of Vγ9Vδ2 T and Car-B7H3-γδT cells in vivo, we established a GBM PDX mouse model by injecting U87-MG-Luc cells into the frontal cortex. In vivo, bioluminescence was performed weekly to monitor tumor size, and then Vγ9Vδ2 T or Car-B7H3-γδT cells or PBS were infused into the lateral cerebral ventricle once a week for two weeks. Bioluminescence analysis showed that tumors were formed in the mouse brain on day 7 after tumor cell injection (Fig. 4A**)**. Both Vγ9Vδ2 T and Car-B7H3-γδT cell treatments significantly suppressed GBM tumor growth in mice compared to the vehicle control (Fig. 4A). Notably, Car-B7H3-γδT cells demonstrated a stronger inhibition of mouse brain tumor growth than Vγ9Vδ2 T cells (*P* < 0.0001) (Fig. 4B). Moreover, the survival of both the Car-B7H3-γδT and Vγ9Vδ2 T groups was significantly longer than that of the control group (44 and 38 days vs. 28 days; both *P* < 0.0001), and the survival of the Car-B7H3-γδT group was longer than that of the Vγ9Vδ2 T group (44 days vs. 38 days, *P* < 0.0001) (Fig. 4C). These data suggest that Car-B7H3-γδT cells demonstrate stronger inhibition of GBM growth and improve prognosis in vivo compared to Vγ9Vδ2 T cells.

### Vγ9Vδ2 T and Car-B7H3-γδT cells infiltrate into GBM tumors in mice after intracranial injection

**Fig. 5:**
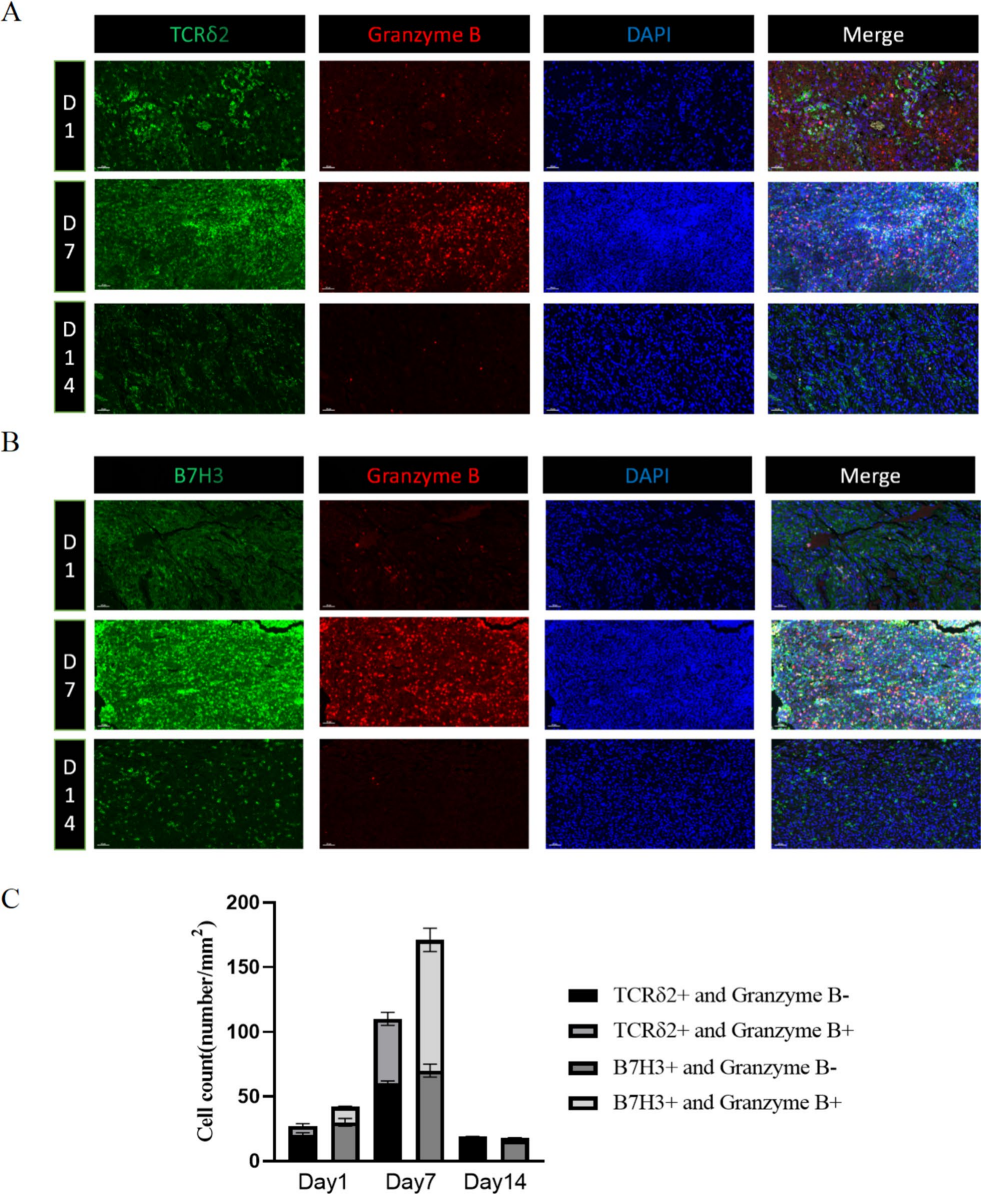
Vγ9Vδ2 T cells and Car-B7H3-γδT cells infiltrated into tumor xenografts in mice Tumor-bearing mice received an intracranial injection of 5 × 10^6^ Vγ9Vδ2 T cells **A** or Car-B7H3-γδT cells **B**, followed by multiplex immunofluorescence staining to detect human TCRδ2, B7H3, and Granzyme B expression in tumor xenografts on day 1, day 7, and day 14 after injection. **C** The percentage of activated Vγ9Vδ2T (TCRδ2^+^ and Granzyme B^+^) cells and Car-B7H3-γδT (B7H3^+^ and Granzyme B^+^) cells (number /mm^2^) in total Vγ9Vδ2 T cells at different time points was analyzed. Data are expressed as the mean ± SD

We injected Vγ9Vδ2T or Car-B7H3-γδT cells into the lateral ventricle of orthotopically implanted mouse models. Opal multi-dimensional fluorescence staining was performed on tumor tissues harvested on day 1, day 7, and day 14 after infusion. TCRδ2 labeled Vγ9Vδ2 T cells, B7-H3 labeled Car-B7H3-γδT cells, and Granzyme B was used to label activated T cells (killing activity) (Fig. 5A, B). On day 1, both Vγ9Vδ2 T and Car-B7H3-γδT cells infiltrated the brain stem tumor of mice, with a small number of cells becoming activated and beginning to play a role in killing. Furthermore, the infiltration and activation of Vγ9Vδ2T (TCRδ2^+^ and Granzyme B^+^) and Car-B7H3-γδT cells (B7H3^+^ and Granzyme B^+^) peaked on day 7, with more activated Car-B7H3-γδT cells infiltrating the tumor tissue. The infiltrations of both cell types decreased by day 14 (Fig. 5C), indicating that patients could receive reinfusion therapy with Vγ9Vδ2T and Car-B7H3-γδT cells every two weeks in a clinical setting.

### Vγ9Vδ2 T and Car-B7H3-γδT therapy is safe against GBM tumors in mice after intracranial injection

We evaluated the safety of Vγ9Vδ2 T cell therapy by injecting low-dose (5 × 10^5^ cells/5 μL PBS) or high-dose (5 × 10^6^ cells/5 μL PBS) Vγ9Vδ2 T cells into the lateral ventricle of healthy NSG mice. H&E staining at 7 days after injection showed that Vγ9Vδ2 T cell therapy did not induce any significant histological changes in the liver, lung, ovary, brain, spleen, kidney, stomach, heart, or uterus of the mice compared to control treatment (Additional file 1: Figure S3A, S3B). Additionally, no significant change was observed in the body weight of the mice within 18 days after low or high dose of Vγ9Vδ2 T cell or Car-B7H3-γδT cell injection (Additional file 1: Figure S4A and S4B). These data suggest that Vγ9Vδ2 T cell therapy is well tolerated in NSG mice. To address the potential for cytokine release syndrome and neurotoxicity from immune cells [34], we measured the serum inflammatory cytokine levels of the mice and found no significant differences among the three groups (*P* > 0.05) (Additional file 1: Figure S4C).

## Discussion

While the current standard therapies for glioma, including surgery, radiotherapy, and chemotherapy, have been employed, the prognosis for patients remains suboptimal. Vγ9Vδ2 T cell immunotherapy offers a promising approach. In this study, we demonstrated that Vγ9Vδ2 T cells derived from healthy human PBMCs inhibited GBM cell proliferation in vitro and suppressed GBM tumor growth in vivo without apparent toxicity to mice, consistent with recent reports [35–37]. However, the response to Vγ9Vδ2 T cell therapy varied greatly among PTCs, leading us to classify them into SAT and WAT groups. We therefore aimed to identify potential indicators associated with the response to Vγ9Vδ2 T cell therapy. IHC staining conducted on matched tumor tissue samples from PTCs in the SAT group confirmed the elevated expression levels of BTN2A1 and BTN3A1. Intriguingly, Vγ9Vδ2 T cells elicited a strong antitumor effect in 23.1% of PTCs, which was significantly associated with higher expression levels of BTN2A1 and BTN3A. These findings suggest that BTN2A1 and BTN3A1 hold promise as potential indicators for the response of GBM patients to Vγ9Vδ2 T cell therapy. To translate this finding from bench to bedside, further data from a larger cohort is required. In addition, a more convenient method to detect BTN2A1 and BTN3A1 expression would be necessary.Dysregulation of the mevalonate pathway is common in tumor cells, leading to the accumulation of both DMAPP and IPP, which could be recognized by Vγ9Vδ2 T cells [38–40]. Our RNA-seq analysis of SAT and WAT tissues further revealed the involvement of lipid metabolism and inflammation-related pathways in the antitumor effect of Vγ9Vδ2 T cells, providing valuable insights for future studies.

Although Vγ9Vδ2 T cells derived from human PBMCs demonstrated good cytotoxicity against gliomas that overexpress BTN2A1/BTN3A1, such overexpression occurred in less than 30% of GBM cases. This led us to explore the use of zoledronate, a bisphosphonate drug that inhibits farnesyl diphosphate synthase to accumulate DMAPP and IPP, or a BTN3A1 agonistic antibody 20.1 to sensitize WAT primary glioma cells to Vγ9Vδ2 T cell killing. Recognizing the clinical limitations of ZOL and BTN3A1 agonistic antibody in glioma, especially given the high B7H3 expression in the majority of GBM cases, we genetically engineered Vγ9Vδ2 T cells into Car-B7H3-γδT cells. Importantly, Car-B7H3-γδT cells can recognize tumors via B7H3-Car and also possess the ability to eliminate tumors through phosphoantigen recognition. Our study showed that Car-B7H3-γδT cells were more potent than unmodified Vγ9Vδ2 T cells at inhibiting GBM cell proliferation in vitro and improving the overall survival of tumor-bearing mice, suggesting that genetic modification of Vγ9Vδ2 T cells may enhance the therapeutic efficacy and clinical outcomes of Vγ9Vδ2 T cell-based immunotherapy for GBM. Although Vγ9Vδ2 T cells derived from human PBMCs demonstrated good cytotoxicity against gliomas that overexpress BTN2A1/BTN3A1, such overexpression occurred in less than 30% of GBM cases. Therefore, Car-B7H3-γδT cells show greater promise as a potential therapeutic agent for GBM treatment compared to unmodified Vγ9Vδ2 T cells.

As γδ T cell numbers and exercise responsiveness decline with age [44], PBMCs from healthy donors aged 18–24 years were used in this study. However, the specific immune status, the blood–brain barrier, and the lack of a classical lymphatic drainage system in the central nervous system (CNS) can hinder the delivery of effector T cells [45]. Clinical trials of adoptive immunotherapy with human T lymphocytes for GBM have employed intravascular, intratumoral, or intraventricular injection [46]. However, intravascular administration may cause cytokine release syndrome and tumor inflammation-associated neurotoxicity, while intratumoral injection may cause tumor hemorrhage or increased cranial pressure [47]. To prevent intracranial hypertension, a small amount of cerebrospinal fluid can be extracted before intracerebroventricular injection. Therefore, we chose to perform the in vivo study using intracerebroventricular injection. Consistent with studies using this injection method [48–50], our study showed that intracerebroventricular injection of Vγ9Vδ2 T cells and Car-B7H3-γδT cells effectively inhibited brain tumor growth in the absence of small molecule phosphate drugs. Moreover, Vγ9Vδ2 T cells and Car-B7H3-γδT cells were well tolerated in mice and could infiltrate into the tumor tissue. Although TCRδ2 expression was barely detectable in tumor tissue at 14 days after treatment, Vγ9Vδ2 T cells and Car-B7H3-γδT cells displayed potent tumor inhibitory effects within 14 days after injection. These data suggest that intracerebroventricular injection of Vγ9Vδ2 T cells and Car-B7H3-γδT cells is clinically feasible for GBM treatment.

In conclusion, the high expression of BTN2A1/BTN3A1 may serve as a potential indicator for pre-screening Vγ9Vδ2 T cells derived from healthy human PBMCs for glioma treatment. The potent antitumor effect of Vγ9Vδ2 T cells on PTCs may be related to lipid metabolism and inflammation-related pathways. Car-B7H3-γδT cells exhibit stronger anti-glioma abilities compared to parental Vγ9Vδ2 T cells in vitro and demonstrated improved prognostic outcomes in tumor-bearing mice. These findings suggest that γδT cells represent a promising therapeutic agent for GBM.

### Supplementary Information

**Additional file 1: Figure S1.** Hematoxylin and eosin staining of xenograft tumor sample from NSG mouse. Magnification 20 ×. Scale bar = 50 µm. **Figure S2. **A Flow cytometry analysis was conducted to detect the expression of BTN2A1 and BTN3A1 in GBM cell lines. B Car-B7H3-γδT cells were obtained by transfecting Vγ9Vδ2 T cells with plasmids containing scFv of the anti-B7-H3 antibody. Flow cytometry analysis was used to determine the purity of Car-B7H3-γδT cells. C Flow cytometry analysis was performed to determine B7-H3 expression in U87-MG, TJ905, and HTB15 cells. The protein expression of B7-H3 was 92.6%, 91.8%, and 93.2% in U-87MG, TJ905, and HTB15, respectively. D Vγ9Vδ2 T or Car-B7H3-γδT cells were incubated with U-87MG-Luc, TJ905-Luc, or HTB15-Luc cells at different effective target (E: T) ratios of 0:1, 0.5:1, 1 :1, or 3:1 in the presence of IL-2. The luciferase activity was measured to determine the cell viability of GBM cells at 18–20 h. E The culture medium was collected. ELISA was performed to measure IFN-γ and TNF-α levels. Data are expressed as the mean ± SD. *P < 0.05, ***P < 0.001, ****P < 0.0001; n = 3. **Figure S3.** Hematoxylin and eosin staining of the liver, lung, ovary, brain, spleen, kidney, stomach, heart, and uterus tissue samples from NSG mice after injection with PBS A or high-dose (5 × 10^6^ cells/5 μL PBS) Vγ9Vδ2 T cells B. Magnification 10 ×. **Figure S4.** Evaluation of Vγ9Vδ2 T and Car-B7H3-γδT cell toxicity. A The body weights of mice were measured at different time points after intraventricular injection of control (PBS), low-dose of Vγ9Vδ2 T cells (5 × 10^5^ cells/5 μL PBS), or high-dose of Vγ9Vδ2 T cells (5 × 10^6^ cells/5 μL PBS). B The body weights of GBM tumor-bearing mice were measured at different time points after intraventricular injection. C Cytokine multiplex assay was carried out to examine mouse serum cytokine alterations at 7 days after T cell therapy. Z-score of each cytokine was calculated as the mean fluorescence intensity. A heatmap of the z-scores ranging from − 3 to 3 was generated. **Table S1.** Basic characteristics of all PTC patients. **Table S2.** **A** Primary antibody used in Multiplex staining. **B** Experimental conditions and procedures of Multiplex staining of TCRδ2 panel. **C**. Experimental conditions and procedures of Multiplex staining of B7H3 panel. **Table S3.** Relationship between different grades of glioma and different responses to Vγ9Vδ2 T therapy.

## Data Availability

All data generated or analysed during this study are included in this published article [and its Additional file information files].

## Abbreviations

GBM: Glioblastoma
WHO: World Health Organization
TCR: T cell receptor
BTN2A1: Butyrophilin subfamily 2 member A1
MICA/B: MHC class I-related chain proteins A and B
CAR: Chimeric antigen receptor
PBMCs: Peripheral blood mononuclear cells
PTCs: Patient-derived tumor cell clusters
EGFRt: Epidermal growth factor receptor
RNA-Seq: RNA sequencing
SAT: Stronger antitumor effect
WAT: Weaker antitumor effect
PBS: Phosphate-buffered saline
IHC: Immunohistochemistry
MIS: Multiplex immunofluorescence staining
DEGs: Differentially expressed genes
GSEA: Gene set enrichment analysis
PBMCs: Peripheral blood mononuclear cells
SD: Standard deviation
H&E: Hematoxylin & eosin
ICV: Lateral cerebral ventricle
ZOL: Zoledronic acid

## References

[1] Louis DN, Perry A, Reifenberger G, von Deimling A, Figarella-Branger D, Cavenee WK, Ohgaki H, Wiestler OD, Kleihues P, Ellison DW (2016). The 2016 World Health Organization classification of tumors of the central nervous system: a summary. Acta Neuropathol.

[2] Ostrom QT, Patil N, Cioffi G, Waite K, Kruchko C, Barnholtz-Sloan JS (2020). CBTRUS statistical report: primary brain and other central nervous system tumors diagnosed in the United States in 2013–2017. Neuro Oncol.

[3] Stupp R, Mason WP, van den Bent MJ, Weller M, Fisher B, Taphoorn MJ, Belanger K, Brandes AA, Marosi C, Bogdahn U (2005). Radiotherapy plus concomitant and adjuvant temozolomide for glioblastoma. N Engl J Med.

[4] Gittleman H, Boscia A, Ostrom QT, Truitt G, Fritz Y, Kruchko C, Barnholtz-Sloan JS (2018). Survivorship in adults with malignant brain and other central nervous system tumor from 2000–2014. Neuro Oncol.

[5] Mitchell DA, Cui X, Schmittling RJ, Sanchez-Perez L, Snyder DJ, Congdon KL, Archer GE, Desjardins A, Friedman AH, Friedman HS (2011). Monoclonal antibody blockade of IL-2 receptor alpha during lymphopenia selectively depletes regulatory T cells in mice and humans. Blood.

[6] Wang QT, Nie Y, Sun SN, Lin T, Han RJ, Jiang J, Li Z, Li JQ, Xiao YP, Fan YY (2020). Tumor-associated antigen-based personalized dendritic cell vaccine in solid tumor patients. Cancer Immunol Immunother.

[7] Weenink B, French PJ, Sillevis Smitt PAE, Debets R, Geurts M (2020). Immunotherapy in glioblastoma: current shortcomings and future perspectives. Cancers.

[8] Bryant NL, Suarez-Cuervo C, Gillespie GY, Markert JM, Nabors LB, Meleth S, Lopez RD, Lamb LS (2009). Characterization and immunotherapeutic potential of gammadelta T-cells in patients with glioblastoma. Neuro Oncol.

[9] Lamb LS (2009). Gammadelta T cells as immune effectors against high-grade gliomas. Immunol Res.

[10] Silva-Santos B, Serre K, Norell H (2015). gammadelta T cells in cancer. Nat Rev Immunol.

[11] Riganti C, Massaia M, Davey MS, Eberl M (2012). Human gammadelta T-cell responses in infection and immunotherapy: common mechanisms, common mediators?. Eur J Immunol.

[12] Rigau M, Ostrouska S, Fulford TS, Johnson DN, Woods K, Ruan Z, McWilliam HEG, Hudson C, Tutuka C, Wheatley AK (2020). Butyrophilin 2A1 is essential for phosphoantigen reactivity by gammadelta T cells. Science.

[13] Karunakaran MM, Willcox CR, Salim M, Paletta D, Fichtner AS, Noll A, Starick L, Nohren A, Begley CR, Berwick KA (2020). Butyrophilin-2A1 directly binds germline-encoded regions of the Vgamma9Vdelta2 TCR and Is essential for phosphoantigen sensing. Immunity.

[14] Starick L, Riano F, Karunakaran MM, Kunzmann V, Li J, Kreiss M, Amslinger S, Scotet E, Olive D, De Libero G, Herrmann T (2017). Butyrophilin 3A (BTN3A, CD277)-specific antibody 20.1 differentially activates Vgamma9Vdelta2 TCR clonotypes and interferes with phosphoantigen activation. Eur J Immunol.

[15] Correia DV, Lopes A, Silva-Santos B (2013). Tumor cell recognition by gammadelta T lymphocytes: T-cell receptor vs NK-cell Receptors. Oncoimmunol.

[16] Chitadze G, Lettau M, Luecke S, Wang T, Janssen O, Furst D, Mytilineos J, Wesch D, Oberg HH, Held-Feindt J, Kabelitz D (2016). NKG2D- and T-cell receptor-dependent lysis of malignant glioma cell lines by human gammadelta T cells: modulation by temozolomide and A disintegrin and metalloproteases 10 and 17 inhibitors. Oncoimmunology.

[17] Friese MA, Platten M, Lutz SZ, Naumann U, Aulwurm S, Bischof F, Buhring HJ, Dichgans J, Rammensee HG, Steinle A, Weller M (2003). MICA/NKG2D-mediated immunogene therapy of experimental gliomas. Cancer Res.

[18] Hsiao CH, Lin X, Barney RJ, Shippy RR, Li J, Vinogradova O, Wiemer DF, Wiemer AJ (2014). Synthesis of a phosphoantigen prodrug that potently activates Vgamma9Vdelta2 T-lymphocytes. Chem Biol.

[19] Safarzadeh Kozani P, Safarzadeh Kozani P, Rahbarizadeh F (2021). Addressing the obstacles of CAR T cell migration in solid tumors: wishing a heavy traffic. Crit Rev Biotechnol.

[20] Dana H, Chalbatani GM, Jalali SA, Mirzaei HR, Grupp SA, Suarez ER, Raposo C, Webster TJ (2021). CAR-T cells: Early successes in blood cancer and challenges in solid tumors. Acta Pharm Sin B.

[21] Rozenbaum M, Meir A, Aharony Y, Itzhaki O, Schachter J, Bank I, Jacoby E, Besser MJ (2020). Gamma-delta CAR-T cells show CAR-directed and independent activity against leukemia. Front Immunol.

[22] Qin VM, D'Souza C, Neeson PJ, Zhu JJ (2021). Chimeric antigen receptor beyond CAR-T Cells. Cancers.

[23] Nehama D, Di Ianni N, Musio S, Du H, Patane M, Pollo B, Finocchiaro G, Park JJH, Dunn DE, Edwards DS (2019). B7-H3-redirected chimeric antigen receptor T cells target glioblastoma and neurospheres. EBioMedicine.

[24] Du H, Hirabayashi K, Ahn S, Kren NP, Montgomery SA, Wang X, Tiruthani K, Mirlekar B, Michaud D, Greene K (2019). antitumor responses in the absence of toxicity in solid tumors by targeting B7–H3 via chimeric antigen receptor T cells. Cancer Cell.

[25] Majzner RG, Theruvath JL, Nellan A, Heitzeneder S, Cui Y, Mount CW, Rietberg SP, Linde MH, Xu P, Rota C (2019). CAR T cells targeting B7–H3, a pan-cancer antigen, demonstrate potent preclinical activity against pediatric solid tumors and brain tumors. Clin Cancer Res.

[26] Kramer K, Kushner BH, Modak S, Pandit-Taskar N, Smith-Jones P, Zanzonico P, Humm JL, Xu H, Wolden SL, Souweidane MM (2010). Compartmental intrathecal radioimmunotherapy: results for treatment for metastatic CNS neuroblastoma. J Neurooncol.

[27] Xu Y, Xiang Z, Alnaggar M, Kouakanou L, Li J, He J, Yang J, Hu Y, Chen Y, Lin L (2021). Allogeneic Vgamma9Vdelta2 T-cell immunotherapy exhibits promising clinical safety and prolongs the survival of patients with late-stage lung or liver cancer. Cell Mol Immunol.

[28] Guedan S, Posey AD, Shaw C, Wing A, Da T, Patel PR, McGettigan SE, Casado-Medrano V, Kawalekar OU, Uribe-Herranz M (2018). Enhancing CAR T cell persistence through ICOS and 4–1BB costimulation. JCI Insight.

[29] Roselli E, Boucher JC, Li G, Kotani H, Spitler K, Reid K, Cervantes EV, Bulliard Y, Tu N, Lee SB (2021). 4–1BB and optimized CD28 co-stimulation enhances function of human mono-specific and bi-specific third-generation CAR T cells. J Immunother Cancer.

[30] Yin S, Xi R, Wu A, Wang S, Li Y, Wang C, Tang L, Xia Y, Yang D, Li J (2020). Patient-derived tumor-like cell clusters for drug testing in cancer therapy. Sci Transl Med.

[31] Cano CE, Pasero C, De Gassart A, Kerneur C, Gabriac M, Fullana M, Granarolo E, Hoet R, Scotet E, Rafia C (2021). BTN2A1, an immune checkpoint targeting Vgamma9Vdelta2 T cell cytotoxicity against malignant cells. Cell Rep.

[32] Vavassori S, Kumar A, Wan GS, Ramanjaneyulu GS, Cavallari M, El Daker S, Beddoe T, Theodossis A, Williams NK, Gostick E (2013). Butyrophilin 3A1 binds phosphorylated antigens and stimulates human gammadelta T cells. Nat Immunol.

[33] Okuno D, Sugiura Y, Sakamoto N, Tagod MSO, Iwasaki M, Noda S, Tamura A, Senju H, Umeyama Y, Yamaguchi H (2020). Comparison of a novel bisphosphonate prodrug and zoledronic acid in the induction of cytotoxicity in human Vgamma2Vdelta2 T cells. Front Immunol.

[34] Theruvath J, Sotillo E, Mount CW, Graef CM, Delaidelli A, Heitzeneder S, Labanieh L, Dhingra S, Leruste A, Majzner RG (2020). Locoregionally administered B7-H3-targeted CAR T cells for treatment of atypical teratoid/rhabdoid tumors. Nat Med.

[35] Choi H, Lee Y, Park SA, Lee JH, Park J, Park JH, Lee HK, Kim TG, Jeun SS, Ahn S (2022). Human allogenic gammadelta T cells kill patient-derived glioblastoma cells expressing high levels of DNAM-1 ligands. Oncoimmunology.

[36] Lee M, Park C, Woo J, Kim J, Kho I, Nam DH, Park WY, Kim YS, Kong DS, Lee HW, Kim TJ (2019). Preferential infiltration of unique Vgamma9Jgamma2-Vdelta2 T cells into glioblastoma multiforme. Front Immunol.

[37] Rosso DA, Rosato M, Iturrizaga J, Gonzalez N, Shiromizu CM, Keitelman IA, Coronel JV, Gomez FD, Amaral MM, Rabadan AT (2021). Glioblastoma cells potentiate the induction of the Th1-like profile in phosphoantigen-stimulated gammadelta T lymphocytes. J Neurooncol.

[38] Zhou X, Gu Y, Xiao H, Kang N, Xie Y, Zhang G, Shi Y, Hu X, Oldfield E, Zhang X, Zhang Y (2017). Combining Vgamma9Vdelta2 T Cells with a Lipophilic Bisphosphonate Efficiently Kills Activated Hepatic Stellate Cells. Front Immunol.

[39] Yang Y, Li L, Yuan L, Zhou X, Duan J, Xiao H, Cai N, Han S, Ma X, Liu W (2019). A Structural change in butyrophilin upon phosphoantigen binding underlies phosphoantigen-mediated Vgamma9Vdelta2 T cell activation. Immunity.

[40] Xia Y, Xie Y, Yu Z, Xiao H, Jiang G, Zhou X, Yang Y, Li X, Zhao M, Li L (2018). The mevalonate pathway is a druggable target for vaccine adjuvant discovery. Cell.

[41] Maggs L, Cattaneo G, Dal AE, Moghaddam AS, Ferrone S (2021). CAR T Cell-based immunotherapy for the treatment of glioblastoma. Front Neurosci.

[42] Lin YJ, Mashouf LA, Lim M (2022). CAR T cell therapy in primary brain tumors: current investigations and the future. Front Immunol.

[43] Digregorio M, Coppieters N, Lombard A, Lumapat PN, Scholtes F, Rogister B (2021). The expression of B7–H3 isoforms in newly diagnosed glioblastoma and recurrence and their functional role. Acta Neuropathol Commun.

[44] Pistillo M, Bigley AB, Spielmann G, LaVoy EC, Morrison MR, Kunz H, Simpson RJ (2013). The effects of age and viral serology on gammadelta T-cell numbers and exercise responsiveness in humans. Cell Immunol.

[45] Bailey SL, Carpentier PA, McMahon EJ, Begolka WS, Miller SD (2006). Innate and adaptive immune responses of the central nervous system. Crit Rev Immunol.

[46] Sloan AE, Dansey R, Zamorano L, Barger G, Hamm C, Diaz F, Baynes R, Wood G (2000). Adoptive immunotherapy in patients with recurrent malignant glioma: preliminary results of using autologous whole-tumor vaccine plus granulocyte-macrophage colony-stimulating factor and adoptive transfer of anti-CD3-activated lymphocytes. Neurosurg Focus.

[47] Majzner RG, Ramakrishna S, Yeom KW, Patel S, Chinnasamy H, Schultz LM, Richards RM, Jiang L, Barsan V, Mancusi R (2022). GD2-CAR T cell therapy for H3K27M-mutated diffuse midline gliomas. Nature.

[48] Jarry U, Joalland N, Chauvin C, Clemenceau B, Pecqueur C, Scotet E (2018). Stereotactic adoptive transfer of cytotoxic immune cells in murine models of orthotopic human glioblastoma multiforme xenografts. J Vis Exp.

[49] Joalland N, Chauvin C, Oliver L, Vallette FM, Pecqueur C, Jarry U, Scotet E (2018). IL-21 increases the reactivity of allogeneic human vgamma9Vdelta2 T cells against primary glioblastoma tumors. J Immunother.

[50] Chauvin C, Joalland N, Perroteau J, Jarry U, Lafrance L, Willem C, Retiere C, Oliver L, Gratas C, Gautreau-Rolland L (2019). NKG2D controls natural reactivity of Vgamma9Vdelta2 T lymphocytes against mesenchymal glioblastoma cells. Clin Cancer Res.

[51] Madhok A, Bhat SA, Philip CS, Sureshbabu SK, Chiplunkar S, Galande S (2021). Transcriptome signature of Vgamma9Vdelta2 T cells treated with phosphoantigens and notch inhibitor reveals interplay between TCR and notch signaling pathways. Front Immunol.

[52] Miyashita M, Shimizu T, Ashihara E, Ukimura O (2021). Strategies to improve the antitumor effect of gammadelta T cell immunotherapy for clinical application. Int J Mol Sci.

[53] Huang C, Xiang Z, Zhang Y, Li Y, Xu J, Zhang H, Zeng Y, Tu W (2021). NKG2D as a cell surface marker on gammadelta-T cells for predicting pregnancy outcomes in patients with unexplained repeated implantation failure. Front Immunol.

[54] Xiang Z, Tu W (2017). Dual face of Vgamma9Vdelta2-T cells in tumor immunology: anti—versus pro-tumoral activities. Front Immunol.

[55] Yang R, Zhao Y, Gu Y, Yang Y, Gao X, Yuan Y, Xiao L, Zhang J, Sun C, Yang H (2020). Isocitrate dehydrogenase 1 mutation enhances 24(S)-hydroxycholesterol production and alters cholesterol homeostasis in glioma. Oncogene.

