# Supplementary material for: B7H3-targeting chimeric antigen receptor modification enhances antitumor effect of Vγ9Vδ2 T cells in glioblastoma
Source: J Transl Med. 2023 Sep 28;21:672. doi: 10.1186/s12967-023-04514-8 (PMC10537973; doi:10.1186/s12967-023-04514-8)
Supplement: Supplementary file 1 — Additional file 1: Figure S1. Hematoxylin and eosin staining of xenograft tumor sample from NSG mouse. Magnification 20 ×. Scale bar = 50 µm. Figure S2. A Flow cytometry analysis was conducted to detect the expression of BTN2A1 and BTN3A1 in GBM cell lines. B Car-B7H3-γδT cells were obtained by transfecting Vγ9Vδ2 T cells with plasmids containing scFv of the anti-B7-H3 antibody. Flow cytometry analysis was used to determine the purity of Car-B7H3-γδT cells. C Flow cytometry analysis was performed to determine B7-H3 expression in U87-MG, TJ905, and HTB15 cells. The protein expression of B7-H3 was 92.6%, 91.8%, and 93.2% in U-87MG, TJ905, and HTB15, respectively. D Vγ9Vδ2 T or Car-B7H3-γδT cells were incubated with U-87MG-Luc, TJ905-Luc, or HTB15-Luc cells at different effective target (E: T) ratios of 0:1, 0.5:1, 1 :1, or 3:1 in the presence of IL-2. The luciferase activity was measured to determine the cell viability of GBM cells at 18–20 h. E The culture medium was collected. ELISA was performed to measure IFN-γ and TNF-α levels. Data are expressed as the mean ± SD. *P < 0.05, ***P < 0.001, ****P < 0.0001; n = 3. Figure S3. Hematoxylin and eosin staining of the liver, lung, ovary, brain, spleen, kidney, stomach, heart, and uterus tissue samples from NSG mice after injection with PBS A or high-dose (5 × 10^6^ cells/5 μL PBS) Vγ9Vδ2 T cells B. Magnification 10 ×. Figure S4. Evaluation of Vγ9Vδ2 T and Car-B7H3-γδT cell toxicity. A The body weights of mice were measured at different time points after intraventricular injection of control (PBS), low-dose of Vγ9Vδ2 T cells (5 × 10^5^ cells/5 μL PBS), or high-dose of Vγ9Vδ2 T cells (5 × 10^6^ cells/5 μL PBS). B The body weights of GBM tumor-bearing mice were measured at different time points after intraventricular injection. C Cytokine multiplex assay was carried out to examine mouse serum cytokine alterations at 7 days after T cell therapy. Z-score of each cytokine was calculated as the mean fluorescence intensity. A heatmap o [file 12967_2023_4514_MOESM1_ESM.zip › Supplementary Materials/Supplemental table 1-3.docx]

**Supplemental table 1: Basic characteristics of all PTC patients**

| Serial number | pathology | WHO grade | IDH mutation status | MGMT methylation status | Primary or recurrence | Preoperative radiotherapy and chemotherapy | steroid exposure prior to resection | average diameter of PTCs（0h） | average diameter of PTCs（8h） | average diameter change of PTCs（8h/0h） | antitumor effect | BTN2A1 | BTN3A1 |
| --- | --- | --- | --- | --- | --- | --- | --- | --- | --- | --- | --- | --- | --- |
| TT-LS | Glioblastoma | IV | wild type | unmethylation | Primary | [without](javascript:;) | [without](javascript:;) | 1.41 | 1.29 | 0.92 | WAT | 10 | 10 |
| TT002 | Anaplastic astrocytoma | III | wild type | unmethylation | Primary | [without](javascript:;) | [without](javascript:;) | 1.06 | 1.37 | 1.30 | WAT | 10 | 20 |
| TT003 | Glioblastoma | IV | wild type | unmethylation | Primary | [without](javascript:;) | [without](javascript:;) | 1.80 | 0.78 | 0.43 | SAT | 80 | 50 |
| TT004 | Anaplastic astrocytoma | III | wild type | unmethylation | Primary | [without](javascript:;) | [without](javascript:;) | 1.00 | 1.10 | 1.10 | WAT | 30 | 20 |
| TT005 | Astrocytoma | II | wild type | methylation | Primary | [without](javascript:;) | [without](javascript:;) | 2.17 | 2.61 | 1.20 | WAT | 20 | 20 |
| TT006 | Astrocytoma | II | wild type | methylation | Primary | [without](javascript:;) | [without](javascript:;) | 1.34 | 1.13 | 0.84 | WAT | 0 | 0 |
| TT007 | Anaplastic astrocytoma | III | [mutant type](javascript:;) | unmethylation | Primary | [without](javascript:;) | [without](javascript:;) | 2.48 | 2.30 | 0.93 | WAT | 10 | 50 |
| TT008 | Astrocytoma | II | [mutant type](javascript:;) | unmethylation | Primary | [without](javascript:;) | [without](javascript:;) | 2.36 | 4.11 | 1.74 | WAT | 10 | 10 |
| TT009 | Oligoastrocytoma | II | [mutant type](javascript:;) | methylation | Primary | [without](javascript:;) | [without](javascript:;) | 1.09 | 1.12 | 1.03 | WAT | 10 | 10 |
| TT010 | Glioblastoma | IV | wild type | methylation | Primary | [without](javascript:;) | [without](javascript:;) | 1.28 | 0.56 | 0.44 | SAT | 50 | 50 |
| TT011 | Oligoastrocytoma | II | [mutant type](javascript:;) | methylation | Primary | [without](javascript:;) | [without](javascript:;) | 2.12 | 3.61 | 1.70 | WAT | 10 | 10 |
| TT012 | Anaplastic astrocytoma | III | wild type | methylation | Primary | [without](javascript:;) | [without](javascript:;) | 1.23 | 0.92 | 0.75 | WAT | 5 | 0 |
| TT013 | Astrocytoma | II | [mutant type](javascript:;) | methylation | Primary | [without](javascript:;) | [without](javascript:;) | 1.00 | 0.73 | 0.73 | WAT | 0 | 0 |
| TT-14 | Glioblastoma | IV | wild type | unmethylation | Primary | [without](javascript:;) | [without](javascript:;) | 2.10 | 1.68 | 0.80 | WAT | 5 | 5 |
| TT-YZJ | Glioblastoma | IV | wild type | unmethylation | Primary | [without](javascript:;) | [without](javascript:;) | 1.25 | 1.00 | 0.80 | WAT | 5 | 5 |
| TT-SX | Glioblastoma | IV | wild type | methylation | Primary | [without](javascript:;) | [without](javascript:;) | 1.21 | 1.08 | 0.89 | WAT | 10 | 0 |
| TT017 | Glioblastoma | IV | wild type | unmethylation | Primary | [without](javascript:;) | [without](javascript:;) | 2.26 | 1.39 | 0.61 | WAT | 6 | 0 |
| TT018 | Glioblastoma | IV | wild type | unmethylation | Primary | [without](javascript:;) | [without](javascript:;) | 2.01 | 1.52 | 0.75 | WAT | 0 | 5 |
| TT019 | Anaplastic astrocytoma | III | wild type | unmethylation | Primary | [without](javascript:;) | [without](javascript:;) | 4.91 | 0.92 | 0.19 | SAT | 50 | 50 |
| TT020 | Oligoastrocytoma | II | [mutant type](javascript:;) | methylation | Primary | [without](javascript:;) | [without](javascript:;) | 1.65 | 0.03 | 0.02 | SAT | 40 | 100 |
| TT021 | Glioblastoma | IV | wild type | unmethylation | Primary | [without](javascript:;) | [without](javascript:;) | 2.18 | 0.07 | 0.03 | SAT | 40 | 80 |
| TT022 | Glioblastoma | IV | wild type | methylation | Primary | [without](javascript:;) | [without](javascript:;) | 1.10 | 0.52 | 0.47 | SAT | 80 | 50 |
| TT-ZLH | Glioblastoma | IV | wild type | unmethylation | Primary | [without](javascript:;) | [without](javascript:;) | 3.03 | 2.97 | 0.98 | WAT | 5 | 5 |
| TT-XJ | Glioblastoma | IV | wild type | methylation | Primary | [without](javascript:;) | [without](javascript:;) | 3.69 | 2.69 | 0.73 | WAT | 10 | 0 |
| TT025 | Glioblastoma | IV | wild type | methylation | Primary | [without](javascript:;) | [without](javascript:;) | 2.96 | 2.69 | 0.91 | WAT | 30 | 50 |
| TT-HCL | Glioblastoma | IV | wild type | unmethylation | Primary | [without](javascript:;) | [without](javascript:;) | 1.02 | 0.96 | 0.94 | WAT | 10 | 0 |

**Supplemental table 2A: Primary antibody used in Multiplex staining**

| **Stain Order** | **Antibody** | **Clone or lot# (host)/Company** | **Dilution** | **Opal dye (Dilution)** | **CITY/Nation** |
| --- | --- | --- | --- | --- | --- |
| **1** | TCRδ2 (H41) | sc-100289 (Mouse)/[Santa Cruz Biotechnology](https://www.baidu.com/link?url=XUhDeOn1Nx7Gb0ArgWzEc8ZDFW59pvqPYeaLkvZiBzZtM9ufu47QL0LByN5k4L7m1RASUaOkdY3_ETlU4bBqI89FjetkYsUlne6nLPfWRkR8PHAdPkQgK6CrMdmsk5ih3FhtsMBNzxDNR8lsXbKNlwpCPmqT6VybasxXSazMyrK&wd=&eqid=d54878c8004588f50000000262f8eaeb) | 1/100 | 540 (1:100) | Cambridge/UK |
| **2** | Granzyme B | Ab4059 (Rabbit)/Abcam | 1/50 | 690 (1:100) | Cambridge/UK |
| **3** | B7H3 | Ab227679 (Rabbit)/Abcam | 1/100 | 540 (1:100) | Cambridge/UK |
| **4** | DAPI | C0060/Solarbio | 1/500 |  | Beijing/China |

**Supplemental table 2B: Experimental conditions and procedures of Multiplex staining of TCRδ2 panel**

| **Antigen** | **TCRδ2** | **Granzyme B** | **DAPI** |
| --- | --- | --- | --- |
| **Antigen retrieval** | AR9  20 min | AR9  20 min |  |
| **Wash** | TBST  10 min | TBST  10 min |  |
| **Blocking** | 10 min  @RT | 10 min  @RT |  |
| **Inc. with Primary antibody** | 60 min  @37℃ | Over night  @4℃ | 10 min  @RT |
| **Wash** | TBST  10 min | TBST  10 min | TBST  10 min |
| **Inc. with Opal HRP Polymer Ms+Rb** | 10 min  @RT | 10 min  @RT |  |
| **Wash** | TBST  10 min | TBST  10 min | Mounting |
| **Inc. with Opal Dye** | opal 540  10 min  @RT | opal 690  10 min  @RT |  |
| **Wash** | TBST  10 min | TBST  10 min |  |

Abbreviations: AR9: Antigen retrieval at pH=9; @; at; RT: at Room temperature; Inc: Incubation; Temp: Temperature; Ms+Rb: Mouse and Rabbit

**Supplemental table 2C: Experimental conditions and procedures of Multiplex staining of B7H3 panel**

| **Antigen** | **B7H3** | **Granzyme B** | **DAPI** |
| --- | --- | --- | --- |
| **Antigen retrieval** | AR9  20 min | AR9  20 min |  |
| **Wash** | TBST  10 min | TBST  10 min |  |
| **Blocking** | 10 min  @RT | 10 min  @RT |  |
| **Inc. with Primary antibody** | 60 min  @37℃ | Over night  @4℃ | 10 min  @RT |
| **Wash** | TBST  10 min | TBST  10 min | TBST  10 min |
| **Inc. with Opal HRP Polymer Ms+Rb** | 10 min  @RT | 10 min  @RT |  |
| **Wash** | TBST  10 min | TBST  10 min | Mounting |
| **Inc. with Opal Dye** | opal 540  10 min  @RT | opal 690  10 min  @RT |  |
| **Wash** | TBST  10 min | TBST  10 min |  |

Abbreviations: AR9: Antigen retrieval at pH=9; @; at; RT: at Room temperature; Inc: Incubation; Temp: Temperature; Ms+Rb: Mouse and Rabbit

**Supplemental table 3: Relationship between different grades of glioma and different responses to Vγ9Vδ2 T therapy**

| WHO grade | Antitumor effect on PTCs | | *P* Value |
| --- | --- | --- | --- |
|  | SAT | WAT |  |
| II | 1 | 6 | >0.05 |
| III | 1 | 4 |  |
| IV | 4 | 10 |  |
